# New Points of Departure for More Global Influenza Vaccine Use

**DOI:** 10.3390/vaccines8030410

**Published:** 2020-07-23

**Authors:** Irina Kiseleva

**Affiliations:** 1 Department of Virology, Federal State Budgetary Scientific Institution, Institute of Experimental Medicine, 197376 St Petersburg, Russia; irina.v.kiseleva@mail.ru; Tel.: +7-812-234-6860; 2 Faculty of Dental Medicine and Medical Technologies, St Petersburg University, 199034 St Petersburg, Russia

**Keywords:** influenza, inactivated vaccines, live attenuated vaccines, next-generation vaccines, synthetic vaccines, cross protectivity, COVID-19 pandemic

## Abstract

Each year, influenza causes a significant acute respiratory disease burden. In addition, influenza pandemics periodically occur. Annual vaccination is the best tool for influenza prevention, but its effectiveness can vary from year to year. The narrow specificity of conventional vaccines and the drug resistance of currently circulating viruses reduce the effectiveness of prophylaxis and treatment and require the development of new broad-spectrum preparations. Furthermore, the challenge of creating a highly effective universal influenza vaccine takes on renewed intensity in the face of the COVID-19 pandemic.

## 1. Introduction

The isolation of the first influenza virus led to the development of the first generation of inactivated and live vaccines. A hundred years after the influenza virus was first isolated, influenza vaccines are still an important influenza prevention tool. Effective vaccines are the key element in influenza control [1]. Vaccination can reduce illness and lessen the severity of infection, especially in at-risk groups [2]. Innovative strategies, such as recombinant technologies, are currently being studied to improve the immunological response [3] and develop universal vaccines [4].

Antiviral treatment shortens the clinical course when given within two days from the onset of disease. Unfortunately, the drug resistance of currently circulating viruses reduces the effectiveness of influenza treatment and dictates the need for the development of new anti-influenza antivirals and new broad-spectrum influenza vaccines.

Those who are interested in the current state of the problem of improving influenza vaccines may find the compressed and clear information desired in the wonderful review article of 12 pages entitled “Efforts to Improve the Seasonal Influenza Vaccine” by Harding and Heaton [4]. In this paper, the authors raise questions of high importance and highlight the different techniques being developed for improving the efficacy of the seasonal influenza vaccination. Different innovative strategies for developing universal vaccines and their respective potential benefits and disadvantages are summarized in [4]. Since the paper [4] was published, the epidemiological situation in the world has changed dramatically. The pandemic invasion of a new SARS-CoV-2 virus began at the end of 2019. In this editorial, our vision of the current situation with flu vaccines is presented.

## 2. Influenza Vaccines: What is Next?

**Figure 1:**
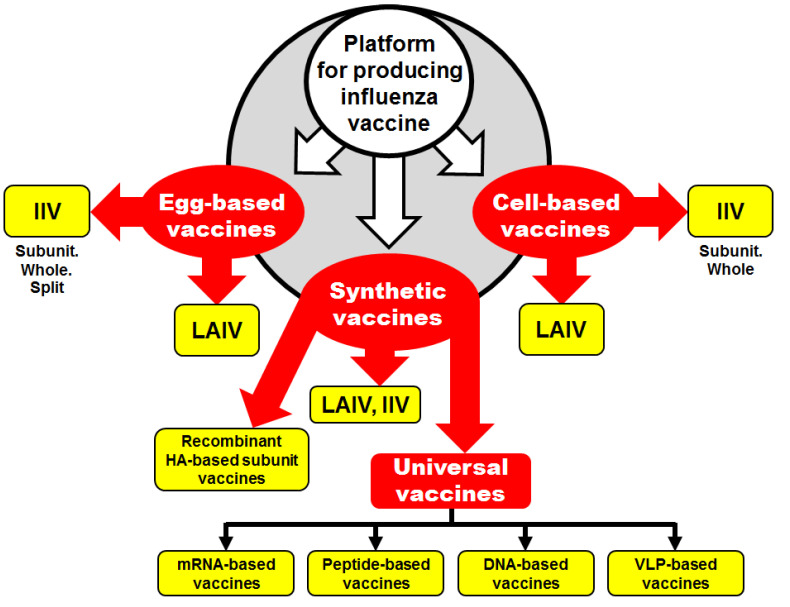
Conventional and novel platforms for influenza vaccine production.

Three parallel approaches for influenza vaccine production are being used by vaccine manufacturers: (i) the production of the conventional egg-derived vaccine; (ii) the production of the cell-culture-derived vaccine; and (iii) the production of synthetic vaccines (the most recent technology) (Figure 1).

### 2.1. Egg-Derived Influenza Vaccines

The majority of influenza vaccines that are currently commercialized by licensed manufacturers are grown in eggs.

Egg-derived live attenuated reassortant influenza vaccines (LAIVs) are generated by the classical co-infection of the epidemiologically relevant wild-type parental strain with a cold-adapted master donor virus, as described elsewhere [5]. As of today, two reassortant LAIVs developed in the US and Russia are available commercially. The first one, licensed in 1987 for the prevention of influenza in people aged 3 years and older as Ultravac (Microgen, Moscow, Russia), is based on cold-adapted master donor viruses (MDVs), A/Leningrad/134/17/57 (H2N2) and B/USSR/60/69; the vaccine candidates are entirely produced by classical reassortment in embryonated chicken eggs. The second one, licensed in 2003 as FluMist (MedImmune, Gaithersburg, MD, USA), is based on cold-adapted master donor viruses, A/Ann Arbor/6/60ca (H2N2) and B/Ann Arbor/1/66ca, and is prescribed for the prevention of influenza in people aged 2–49 years old. The reassortants to be included in FluMist are generated by reverse genetics, with the same 6:2 genome composition as the classical egg-derived vaccine Ultravac.

The quadrivalent LAIV FluMist (MedImmune, Gaithersburg, MD, USA) is based on the A/Ann Arbor/6/60ca and B/Ann Arbor/1/66ca MDVs and is licensed in the US, Canada, and Europe. Trivalent LAIV Ultravac (Microgen, Moscow, Russia) is based on the A/Leningrad/134/17/57 and B/USSR/60/69 MDVs and is licensed in Russia, India, and China.

Inactivated egg-derived vaccines (IIVs) are the most commonly used preparations for influenza prophylaxis. The licensed seasonal inactivated hemagglutinin-based influenza vaccines include four types: (i) split virus, (ii) subunit, (iii) whole-virus inactivated, and (iv) recombinant hemagglutinin-based protein (Figure 1). Recently, numerous advances have been made in the development of IIVs to replace inactivated whole-virus vaccines with split or subunit vaccines which comprise less reactogenic alternatives [6].

### 2.2. Cell-Culture-Derived Influenza Vaccine

The use of tissue culture as a substrate could make influenza vaccine manufacturing independent of the global egg supply and enable an easy scaling up of the production process. The WHO made the first recommendations on the composition of influenza virus vaccines based on cell-isolated reference viruses in 2018 [7]. Compared to egg-based technology, cell-culture-derived influenza vaccines reduce the vaccine production time and risk of contamination during production, are safe for those with an allergy to eggs, and an animal component-free production is feasible [8]. Furthermore, egg passaging might induce adaptive changes for growth in eggs.

Two approaches for the development of cell-based vaccines can be used: (i) some steps of the vaccine preparation are conducted in eggs and the final steps are performed in cells, or (ii) all steps of the vaccine preparation are conducted in cells (Figure 1). The first approach has one serious disadvantage, in that the vaccine viruses contain egg-adapted mutations which may alter the virus antigenicity. However, problems with unwanted egg-adapted mutations can be solved by reverse genetics approaches. The second approach is a more logical one, because vaccine candidates are initially free of egg-adapted mutations.

In 2012, the United States Food and Drug Administration announced the approval of the first seasonal influenza cell-based subunit inactivated influenza vaccine, Flucelvax [9]. Another vaccine, so-called ΔNS1, can be conditionally attributed to live attenuated cell-based vaccines [10]. Its attenuation was attained using a novel attenuation approach involving the deletion of the NS1 gene encoding the multifunctional nonstructural protein, which counteracts the interferon-mediated antiviral response.

### 2.3. Synthetic Influenza Vaccines

Major efforts to improve the approaches for vaccine development are underway. As can be seen from Figure 1, the development of synthetic influenza vaccines is the third platform for influenza vaccine production. Several novel technologies that may improve the production process have been described recently. These technologies could make possible the development of eliciting long-lasting and broadly cross-reactive universal influenza vaccines (UIVs) without the need for eggs [11].

With the development of plasmid-based reverse genetics techniques [12], it is now possible to engineer recombinant influenza viruses entirely from full-length complementary DNA copies of the viral genome by the transfection of vaccine-approved susceptible cells. The gene synthesis technique combined with reverse genetics approaches allows the generation of IIV and LAIV 6:2 vaccine candidates within short time frames.

Recombinant HA-based protein technology may help to solve many problems associated with the use of chicken embryos, such as the low affinity of current H3N2 viruses to eggs, and the necessity of speeding up the vaccine development process. However, the low immunogenicity of recombinant HA-based subunit vaccines necessitates repeated vaccination and the use of adjuvants.

An ideal true UIV can induce a cross-protective, broadly neutralizing immune response against conserved viral antigens; offer a combination of protection from antigenic drift/shift; and confer lifelong immunity. It is effective against all influenza A and B virus subtypes/lineages regardless of any mutations in hemagglutinin (HA) and/or neuraminidase (NA). To design this type of vaccine, the highly conserved epitopes present in HA, NA, or M2 and internal proteins should be targeted to induce cross-protective antibodies and T-cells. The development of a UIV can be significantly facilitated by newly developed platform technologies such as multi-epitopes, virus-like particles (VLP), and DNA- and mRNA-based vaccines.

Over 20 different approaches are described as strategies for the development of UIVs, such as HA-targeting vaccines (chimeric, mosaic, and headless HAs, and mosaic nanoparticles), M2-targeting vaccines, internal protein-targeting vaccines, and NA-targeting vaccines. These approaches include recombinant, protein-based, and virus-like particle vaccines; nucleic-acid-based vaccines; recombinant bacterial and viral vector-based vaccines; M2 ectodomain-based vaccines; HA stalk-domain-based vaccines; NA stalk-domain-based vaccines; etc. [4].

DNA-based vaccines are created by incorporating sequences coding for influenza proteins into a DNA bacterial plasmid. The advantage of DNA vaccines is that the production process starts with the nucleotide sequence of HA, which is artificially synthesized and introduced into a productive context that is completely independent of the whole virus [4]. Influenza DNA vaccines can induce broad-spectrum protective immune responses in various animal models. DNA-plasmid vaccine technology provides a rapid, highly scalable, and safe alternative to conventional influenza vaccine production [13]. However, the levels of cell-mediated and humoral responses induced by the introduction of a DNA vaccine are often insufficient for the development of immunity against pathogens and are typically used with adjuvants to enhance immunogenicity.

Another nucleic-acid-based vaccine platform, namely mRNA-based technology, is very attractive, since it harbors only components required for protein expression. RNA is rapidly degraded and does not interact with the host genome [13]. Thus, mRNA-based vaccines offer safety advantages in comparison to DNA-based vaccines. RNA-based vaccines promise a quick, strain-independent production with high yields from a small manufacturing footprint. Two types of RNA-based influenza vaccines are currently being developed: (i) self-amplifying and (ii) nonreplicating mRNA vaccines [14]. Modified mRNA vaccines encoding HA of avian influenza viruses formulated with lipid nanoparticles generated robust immune responses in animal models, protected them from lethal challenge infection, and were safe and immunogenic in humans [15].

Peptide-based UIVs have involved highly conserved single or multi-epitope small peptides. The design of these vaccines is based on a reduction in disease severity rather than the prevention of infection [6]. When there are no protective antibody responses to newly evolved influenza viruses, T-cells could provide heterosubtypic immunity to various influenza virus subtypes and even unrelated viruses due to conserved peptide homology. Several vaccine candidates based on an *Escherichia coli*-expressed fusion peptide containing different epitopes and composed of single or multiple B- or T-cell epitopes have been tested in clinical trials and were found to be safe and induced a cellular immune response [16].

Virus-like particles are noninfectious particles that closely mimic genuine viruses in antigenic structures. VLPs are obtained from the self-assembly of viral structural proteins in different cell lines (plant, insect, or mammalian cells) without the viral genome [17]. The performance of an insect-cell-derived influenza H1N1pdm09 VLP vaccine was successfully demonstrated in clinical trials in adult volunteers and showed the persistence of antibodies for up to 24 months after vaccination [18].

## 3. Cross-Protection Potency of Influenza Vaccines

**Figure 2:**
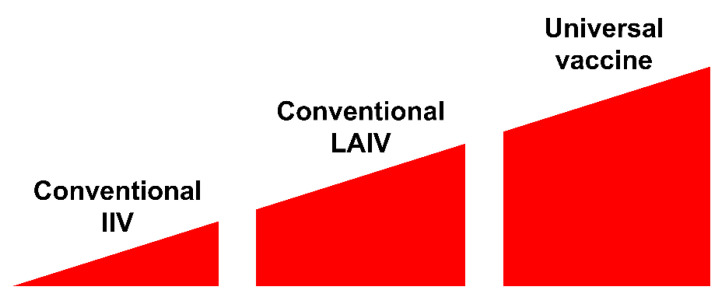
Cross-protective efficacy of different types of influenza vaccine.

Conventional IIVs primarily induce virus-specific adaptive antibody responses—i.e., they are potently strain-specific and poorly effective against mismatched viruses—and small antigenic changes have been associated with a loss in protection [13]. In contrast to traditional inactivated vaccines, LAIVs are capable of inducing broad-spectrum and long-lasting immune responses, making them an attractive option for pandemic preparedness, especially in low-income countries with very high population densities (Figure 2).

The most attractive and promising influenza vaccines are the UIVs, which are designed to induce cross-protective, broadly neutralizing immunity against conserved epitopes of the influenza virus (Figure 2). Recent scientific progress in the development of the potential next-generation UIV is tremendous. UIVs are currently under development in laboratories worldwide. Dozens of research and review articles have been published in recent years by scientists from different countries all over the world [13,14,15,16,17,18,19,20,21,22,23,24,25,26,27,28]. Calls to develop a highly effective universal influenza vaccine may be increased during the COVID-19 pandemic, due to the high potential for the co-circulation of two significant respiratory pathogens.

## 4. The Possible Beneficial Effect of Bacillus Calmette-Guérin (BCG) and Influenza Vaccinations on the Severity of Illness after COVID-19 Infection

An unprecedented effort in developing a COVID-19 vaccine is ongoing. Almost 150 teams all over the world are working hard, with a diverse set of strategies and platforms, to develop a potent and harmless vaccine against COVID-19. As of 14 July 2020, 140 vaccines are in preclinical evaluation and 23 are in clinical trial [29]. However, some caution is prudent, and the gains that have been recently made in understanding the interactions between the host immune system and viruses should be fully exploited [30]. We have to be mindful that the negative consequence of a coronavirus vaccination is the potential for vaccine-induced disease enhancement [31]. Vaccination with a vaccine against COVID-19 followed by infection with wild-type SARS-CoV-2 virus may induce severe disease [32], including a potentially dangerous life-threatening event known as a “cytokine storm”, which is an uncontrolled over-production of the soluble markers of inflammation [33].

As no licensed COVID-19 vaccine is available yet, scientists have put forward theories for which of the available immunobiological preparations may be used in the fight against this disease. In particular, a possible role of vaccination with BCG in preventing novel coronavirus infection has been discussed [34]. Based on the hypothesis that BCG can enhance the reactivity of the innate immune system, some researchers have speculated that the BCG vaccine may be used as a preventive and/or therapeutic measure against COVID-19. Nonetheless, the results have been conflicting, and the official position of the WHO is that there is no evidence that the BCG vaccine protects people against infection with the COVID-19 virus [35].

Besides COVID-19, influenza remains an ever-present cause of disease and death around the globe [36]. The co-circulation of more than one pathogen in one host is common across viruses. Thus, patients may be simultaneously co-infected with SARS-CoV-2 and the influenza virus, leading to a higher risk of developing poor outcomes. Researchers are debating whether the influenza vaccine could be used as an alternative approach until a potent and harmless vaccine against COVID-19 is developed [37]. Given the potential of a global influenza–COVID-19 co-infection [38,39], it has been suggested that the influenza vaccination could be used to indirectly control COVID-19 [37,40]. Of course, the influenza vaccine will not fully protect against coronavirus infection. However, vaccination with a proven and safe influenza vaccine may affect the incidence of the mixed influenza–COVID-19 infection and thus reduce the severity of coronavirus infection. From this point of view, the LAIV might be the most suitable among existing licensed influenza vaccines because of its broad-spectrum potency (Figure 2). A second wave of COVID-19 is expected in parallel with the start of the influenza season, and some have suggested that this fall’s influenza vaccine for larger groups of the population could be recommended to simplify clinicians’ work [39].

## 5. Conclusions

A century after the influenza virus was first isolated, influenza vaccines are still an important influenza prevention tool. However, as Nachbagauer and Palese suggest [11], conventional seasonal influenza vaccines “do not provide sufficient protection to alleviate the annual impact of influenza and cannot confer protection against potentially pandemic influenza viruses.” A number of reasons contribute to the insufficient control of influenza, leading to our failure to eradicate influenza worldwide—e.g., the genetic flexibility of influenza viruses, the rapid evolution of the viral genome, escape immunity, the global spread of drug-resistant influenza strains, not always having a sufficient level of vaccination coverage, etc. [41]. In this situation, the development of highly effective and broad-spectrum vaccines is important. As of today, among licensed vaccines, the privilege may belong to LAIVs as preparations that induce broad-spectrum and long-lasting immune responses.

Several novel technologies that may improve the vaccine production process have been described recently. These disruptive technologies could bring dramatic changes in the current system of influenza vaccine production by the development of protective, long-lasting, and broadly cross-reactive universal influenza vaccines [11]. It is most likely that UIVs are the future of immunization. A major area of concern for the development of UIVs is their effectiveness, and only future research will reveal the most promising and potent strategy.

A similar situation has been observed with the development of vaccines against COVID-19. Over 160 vaccines against COVID-19 are currently under development or in clinical trials [29]. Only a few of them will reach the stage of a mass immunization campaign.

Today, everyone is wondering how long it will take to develop a COVID-19 vaccine. In the face of the COVID-19 pandemic, the necessity for developing a highly effective universal influenza vaccine may be increased. Given the fact of a global spread of influenza–COVID-19 mixed infection, immunization with the flu vaccine may reduce the severity of the mixed influenza–COVID-19 infection [37]. In the absence of a vaccine against COVID-19, a preventive influenza vaccination could become an important parameter in the epidemiological control strategy for COVID-19. We cannot exclude the possibility that, as live or universal flu vaccines have a wide range of cross-immunity, they may somehow also protect against COVID-19, but so far there is no evidence.

Some believe that the complete control of influenza viruses seems impossible [42]. Nevertheless, others feel strongly that such a day will come and that human influenza will be defeated or, at least, effectively controlled.
